# Immunoproteasome Overexpression Underlies the Pathogenesis of Thyroid Oncocytes and Primary Hypothyroidism: Studies in Humans and Mice

**DOI:** 10.1371/journal.pone.0007857

**Published:** 2009-11-17

**Authors:** Hiroaki J. Kimura, Cindy Y. Chen, Shey-Cherng Tzou, Roberto Rocchi, Melissa A. Landek-Salgado, Koichi Suzuki, Miho Kimura, Noel R. Rose, Patrizio Caturegli

**Affiliations:** 1 Department of Pathology, The Johns Hopkins School of Medicine, Baltimore, Maryland, United States of America; 2 Department of Bioregulation, Leprosy Research Center, National Institute of Infectious Diseases, Tokyo, Japan; 3 Feinstone Department of Molecular Microbiology and Immunology, The Johns Hopkins Bloomberg School of Public Health, Baltimore, Maryland, United States of America; BMSI-A*STAR, Singapore

## Abstract

**Background:**

Oncocytes of the thyroid gland (Hürthle cells) are found in tumors and autoimmune diseases. They have a unique appearance characterized by abundant granular eosinophilic cytoplasm and hyperchromatic nucleus. Their pathogenesis has remained, thus far, unknown.

**Methodology/Principal Findings:**

Using transgenic mice chronically expressing IFNγ in thyroid gland, we showed changes in the thyroid follicular epithelium reminiscent of the human oncocyte. Transcriptome analysis comparing transgenic to wild type thyrocytes revealed increased levels of immunoproteasome subunits like LMP2 in transgenics, suggesting an important role of the immunoproteasome in oncocyte pathogenesis. Pharmacologic blockade of the proteasome, in fact, ameliorated the oncocytic phenotype. Genetic deletion of LMP2 subunit prevented the development of the oncocytic phenotype and primary hypothyroidism. LMP2 was also found expressed in oncocytes from patients with Hashimoto thyroiditis and Hürthle cell tumors.

**Conclusions/Significance:**

In summary, we report that oncocytes are the result of an increased immunoproteasome expression secondary to a chronic inflammatory milieu, and suggest LMP2 as a novel therapeutic target for the treatment of oncocytic lesions and autoimmune hypothyroidism.

## Introduction

Oncocytes and lymphocytic infiltration are the pathologic hallmarks of Hashimoto thyroiditis, one of the most prevalent autoimmune diseases [1]. Thyroid oncocytes, also known as Hürthle cells, Askanazy cells, or oxyphilic cells, derive from the thyroid follicular cell and are characterized by an abundant, eosinophilic and granular cytoplasm (the consequence of an increased number of mitochondria) and a large, hyperchromatic nucleus with prominent nucleoli [2], [3]. Thyroid oncocytes are found not only in Hashimoto thyroiditis but also in long-standing Graves disease, multinodular goiter, benign thyroid neoplasms (Hürthle cell adenoma and granular cell tumor), and malignant thyroid neoplasms (Hürthle cell carcinoma, oncocytic variant of papillary carcinoma, Warthin-like variant of papillary carcinoma, and tall cell variant of papillary carcinoma) [4]. Oncocytes, however, are not limited to the thyroid gland. They are also found in other organs like kidneys and salivary glands.

The pathogenesis of oncocytes has remained overall unknown. We have previously developed a mouse model of Hashimoto thyroiditis based on the chronic production of interferon-gamma (IFNγ) by the thyroid follicular cell via transgenesis [5], [6]. These mice develop primary hypothyroidism as the direct consequence of a chronic, cytokine-mediated, inhibition of thyroid function, a phenotype that is present also in the absence of infiltrating lymphocytes (obtained by crossing the transgenic mice to RAG deficient mice) [5]. Interestingly, the thyrocytes of IFNγ transgenic mice assume a morphology resembling that of the human oncocyte.

This paper highlights the role of the immunoproteasome in the pathogenesis of thyroid oncocytes and hypothyroidism. The proteasome is a large barrel-shaped, proteolytic complex present in the nucleus and cytoplasm of all cells to degrade proteins marked with ubiquitin [7]. It is implicated in a number of fundamental cellular activities such as the processing of antigens presented by MHC class I molecules [8]. The constitutive proteasome is made of a 20S catalytic core closed at both ends by a 19S cap. The core is formed by four stacked rings, each made of seven subunits: two alpha rings are on the sides and allow the entrance of only denatured proteins; two beta rings are in the center and perform the protease activity, reducing proteins to small peptides of 3–23 amino acids. Proteolysis is carried out by three beta subunits, β1, β2, and β5, which harbor postglutamyl peptide hydrolytic-like, trypsin-like, and chymotrypsin-like activities, respectively. When the cell is stimulated by IFNγ or other inflammatory stimulants like tumor necrosis factor α, three subunits in the beta rings and two in the cap are replaced by new subunits called iβ1 (or LMP2), iβ2 (or LMP10), iβ5 (or LMP7), PA28α and PA28β, overall giving to the complex the name of “immunoproteasome”. The iβ1 and iβ2 subunits must be assembled together, and similarly PA28α and PA28β, but variations in immunoproteasome do exist. The genes coding for LMP subunits are embedded within the class II region of the MHC locus on chromosome 6, and are therefore in strong linkage disequilibrium with the classical MHC class II molecules (DP, DR, DQ) that have been associated with numerous autoimmune diseases. In this paper we show that LMP2 is required for the pathogenesis of thyroid oncocytes and hypothyroidism.

## Results

### Expression of IFNγ in the Thyroid Transforms Normal Thyrocytes into Oncocytes

**Figure 1:**
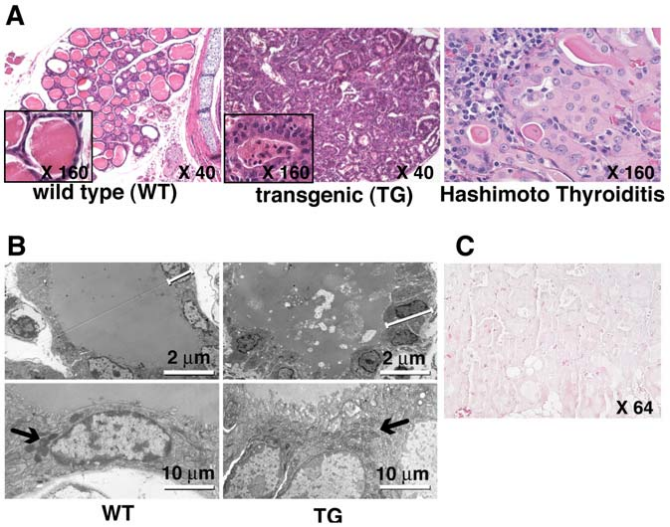
Thyroid histopathology. (A) Thyroids from *thyr*-IFNγ transgenic mice (middle panel) show a disrupted architecture, with scanty colloid and thickened thyrocytes (inset), an appearance markedly different from that of wild type thyroids (left panel). Thyrocytes from *thyr*-IFNγ transgenic mice resemble human Hürthle cells, with eosinophilic and granular cytoplasm and hyperchromatic nucleus, mimicking the features of Hürthle cells from a patient with Hashimoto thyroiditis (right panel). (B) By electron microscopy, *thyr*-IFNγ transgenic thyrocytes appear taller (upper right panel) than wild type thyrocytes (upper left panel), and contain more mitochondria (lower right panel, arrow) than wild type (lower left panel). (C) No apoptosis was detected by TUNEL staining in *thyr*-IFNγ transgenic thyrocytes.

The thyrocytes of *thyr*-IFNγ transgenic mice crowded along a scanty colloidal space (Figure 1A, middle panel), assuming a tall and polygonal shape (Figure 1A, middle panel inset). Their cytoplasm was granular and more eosinophilic than that of wild type thyrocytes (Figure 1A, left panel), and the nucleus enlarged with a prominent nucleolus, resembling the appearance of the oncocytes (Hürthle cells) found in patients with Hashimoto thyroiditis (Figure 1A, right panel). These cellular features were confirmed by electron microscopy, which also revealed an increased number and size of mitochondria in transgenic thyrocytes (Figure 1B). Apoptosis of thyrocytes was not detected by TUNEL in *thyr*-IFNγ transgenic mice (Figure 1C).

### Immunoproteasome Subunits Are Highly Expressed in Oncocyte-Like IFNγ Transgenic Thyrocytes

**Figure 2:**
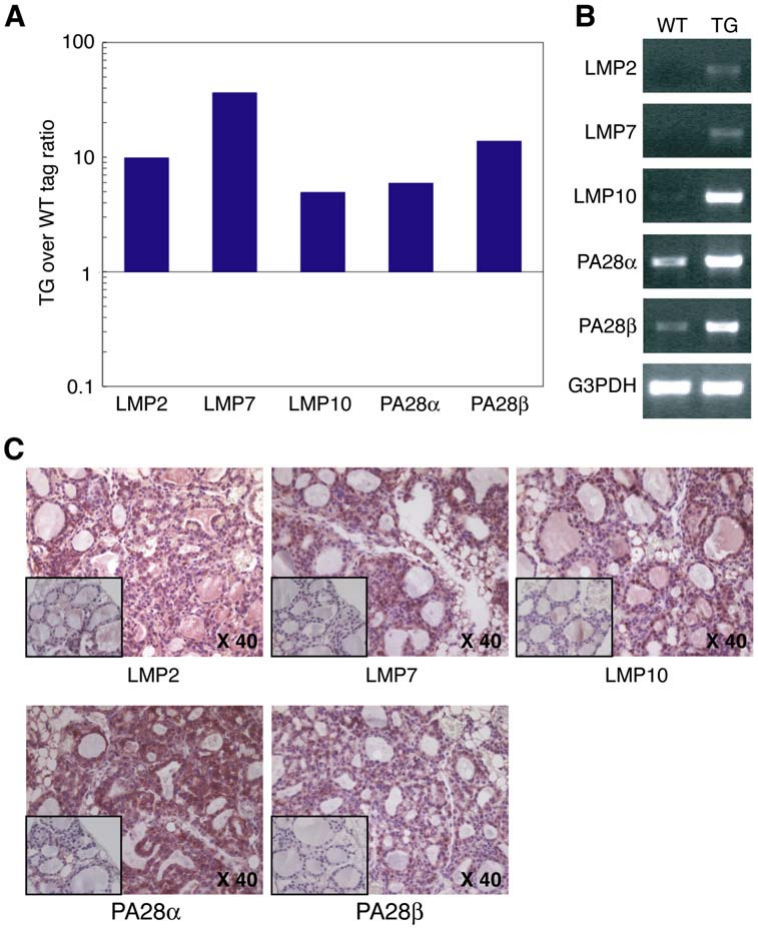
Thyroid expression of immunoproteasome subunits. (A) Immunoproteasome SAGE tag numbers, comparing thyr-IFNγ transgenic to wild type thyrocytes. (B) Immunoproteasome mRNA levels by semi-quantitative RT-PCR, comparing thyr-IFNγ transgenic to wild type thyrocytes. G3PDH is used as housekeeping control. (C) Immunoproteasome protein expression by immunohistochemistry: all subunits are increased in thyr-IFNγ transgenic thyroids when compared to wild type thyroids (inset).

To characterize at the molecular level the pathologic changes seen in *thyr*-IFNγ transgenic thyrocytes, we used long serial analysis of gene expression (Long SAGE) and compared gene expression in transgenic and wild type isolated thyrocytes. A total of 43,718 and 43,908 tags were collected from the transgenic and wild type thyroid libraries, respectively. Major histocompatibility complex (MHC) genes were the most highly expressed genes in transgenic thyrocytes (rank 1, 5, 8 and 10 in Table S1 and 1, 2 and 3 in Table S2), whereas thyroglobulin dominated in wild type controls (rank 1, 2, 6, 7, and 9 in Table S3 and 1, 2, 5 and 7 in Table S4). Comparison of expression tags between *thyr*-IFNγ transgenic and wild type thyrocytes (Table 1) revealed that, following the MHC genes, immunoproteasome subunits were most abundantly expressed in transgenics. In particular, the beta subunit type 8 (also known as LMP7) was increased 36 fold (Table 1); then, the remaining four immunoproteasome subunits were increased as follows: 14 fold for PA28β, 10 fold for beta subunit type 9 (LMP2), 6 fold for PA28α, and 5 fold for beta subunit type 10 (LMP10) (Figure 2A). The complete list of expressed tags in the two thyroid libraries can be downloaded from the GEO website (see Material and methods).

**Table 1:** Comparison of gene expression by SAGE in thyrocytes isolated from *thyr*-IFNγ transgenic (TG) or wild type (WT) mice (shows the genes that displayed the highest TG over WT ratio).

Rank	Gene Description	Unigene ID	Fold change (TG over WT)
1	H2-K1 MHC class I heavy chain precursor (H-2K(d))	Mm.16771	325
	MHC class I (B7.2) cell surface antigen mRNA, 3′ end	Mm.380380	
2	Ecotropic viral integration site 2a	Mm.164948	182
	Ia-associated invariant chain	Mm.276499	
	SH2 domain containing 4A	Mm.40974	
3	Histocompatibility 2, Q region locus 8 (H2-Q8), mRNA	Mm.296901	72
	Transcribed locus	Mm.34421	
4	Connective tissue growth factor	Mm.1810	51
5	Histocompatibility 2, class II antigen E beta (H2-Eb1)	Mm.22564	38
	C76746 Adult male epididymis cDNA, RIKEN full-length enriched library, clone:9230104M02 product	Mm.358798	
6	Proteasome (prosome, macropain) subunit, beta type 8 (large multifunctional peptidase 7: LMP7)	Mm.180191	36
7	Integral membrane protein 2B	Mm.4266	32
8	Ribosomal protein L36	Mm.11376	21
	Transcribed locus, strongly similar to NP_071949.1 ribosomal protein L36 [Rattus norvegicus]	Mm.296314	
	Rpl36 Adult male heart cDNA, 60S RIBOSOMAL PROTEIN L36 homolog	Mm.371604	
	Rpl36 Sulfatase modifying factor 1 (Sumf1)	Mm.379094	
9	Transcribed locus	Mm.234875	20
	RIKEN cDNA 0610037M15 gene	Mm.221293	
	Histocompatibility 2, Q region locus 8 (H2-Q8)	Mm.296901	
	Histocompatibility 2, Q region locus 1	Mm.327075	
10	Hydroxysteroid (17-beta) dehydrogenase 4	Mm.277857	19
	CD63 antigen	Mm.371552	

Long SAGE results were confirmed by reverse transcriptase PCR and immunohistochemistry. The mRNA levels of all five immunoproteasome subunits were significantly over-represented in IFNγ transgenic compared to wild type thyrocytes (Figure 2B). Similarly, the protein levels of LMP2, LMP7, LMP10, PA28α and PA28β were more abundant in IFNγ transgenics than in controls (Figure 2C). The increased expression of immunoproteasome subunits was directly dependent upon IFNγ signaling because it disappeared when *thyr-*IFNγ transgenic mice were crossed to mice lacking Stat1 (Figure S1A) and could be reproduced in cultured Fisher rat thyroid cells exposed to IFNγ (Figure S1B). Based on these findings we hypothesize that the oncocytic phenotype is a consequence of increased immunoproteasome expression, secondary to chronic IFNγ stimulation in our system.

### Pharmacologic Inhibition of the Proteasome Ameliorates the Oncocytic Phenotype

**Figure 3:**
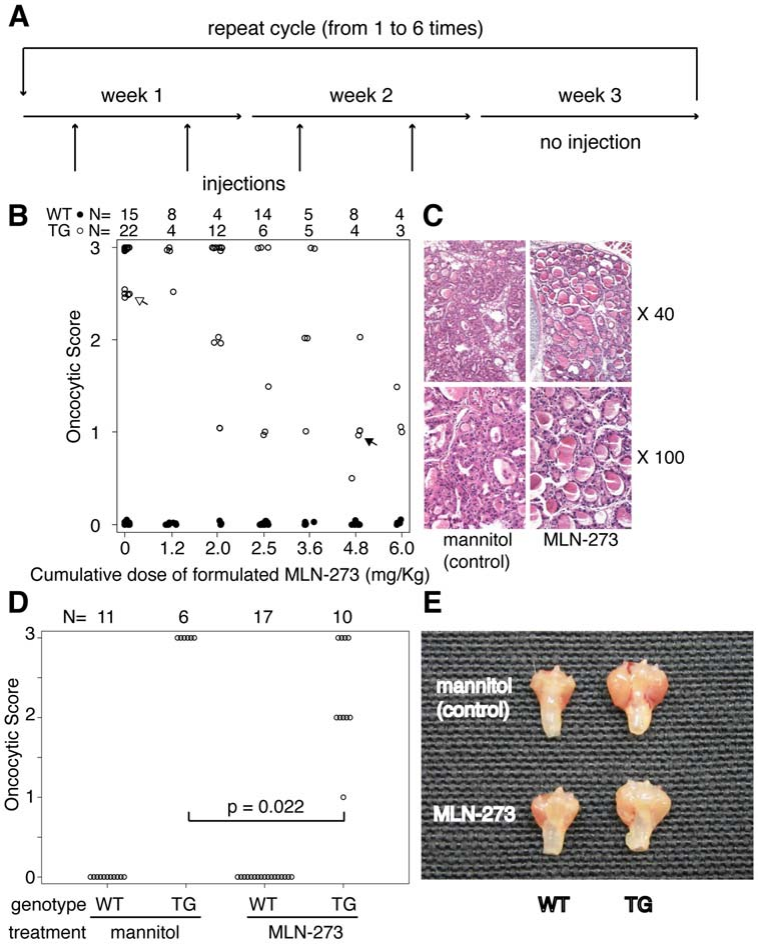
Pharmacologic inhibition of proteasome using MLN-273. (A) Schedule of MLN-273 administration. (B) The oncocytic phenotype typical of *thyr*-IFNγ transgenic mice (open circles) improved upon MLN-273 administration in a dose-dependent fashion, beginning at cumulative doses of 3.6 mg/Kg of body weight. No morphological changes were observed in *thyr*-IFNγ transgenic treated with mannitol control (open circles, dose 0), or in wild type littermates (closed circles). (C) Thyroid morphology at 40X and 100X of a *thyr*-IFNγ transgenic mouse treated with 4.8 mg/Kg MLN-273 (closed arrow in Figure 2B), and another treated with 1.2 mg/Kg MLN-273. (D) Administration of 12 injections of 0.3 mg/Kg MLN-273 significantly improved the oncocytic phenotype of *thyr*-IFNγ transgenic mice. (E) The improvement was also evident macroscopically by a decrease in goiter size.

To assess pharmacologically whether proteasome inhibition influenced the thyroid disease typical of *thyr*-IFNγ transgenic mice, we used MLN-273, a compound manufactured by Millennium Pharmaceuticals, Inc. (Cambridge, MA) similar to Velcade, with a inhibitory activity against purified proteasome of 0.2 nM [9]. Mice (N = 114∶58 wild type and 56 transgenics) were first treated with increasing doses of formulated MLN-273 (0, 0.1, 0.2, 0.3, 0.4, and 0.5 mg per Kg of mouse weight), following an administration cycle of two injections per week for two weeks and one week of rest (Figure 3A). Injections were begun during the first week of life (typically on day 3), and ranged from a minimum of 4 to a maximum of 24 (1 to 6 cycles). MLN-273 ameliorated the oncocytic morphology in *thyr*-IFNγ transgenic mice (Figure 3B, open circles), starting at cumulative doses of 3.6 mg/Kg and reaching a maximum at 6.0 mg/Kg (Figure 3B). An example of improved morphology is shown in Figure 3C, which compares the thyroid appearance of a mouse receiving 6 mg (closed arrow in Figure 3B) to that of a mouse receiving 1.2 mg (open arrow in Figure 3B). No effect on thyroid morphology was seen in wild type mice receiving various doses of MLN-273 (Figure 3B, closed circles), or the mannitol control (Figure 3B, open circles at 0 mg/Kg). Serum T4 levels did not significantly improve upon MLN-273 treatment (data not shown).

We repeated the experiment using a dose of 0.3 mg/Kg for 12 injections (cumulative dose of 3.6 mg/Kg) in 44 mice (28 wild type and 16 transgenic). The oncocytic phenotype improved in *thyr*-IFNγ transgenic mice receiving MLN-273 compared to those receiving mannitol (Figure 3D, p = 0.022). The improvement was also evident by an approximate 6-fold reduction in the weight of thyroid-tracheal blocks (Figure 3E).

### Genetic Deletion of LMP2, but Not LMP7, Corrects the Oncocytic Phenotype

**Figure 4:**
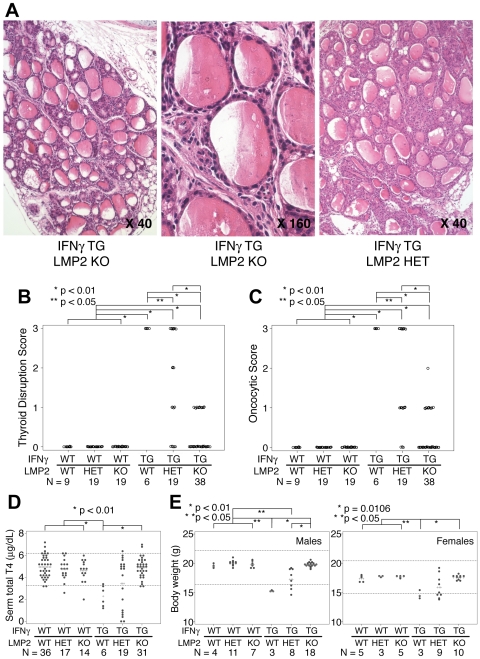
Genetic deletion of LMP2 ameliorates thyroid morphology and function. (A) Thyroid architecture of a *thyr*-IFNγ transgenic-LMP2 knockout mouse at 40X (left panel) and 160X magnification (middle panel). Thyroid architecture of a *thyr*-IFNγ transgenic-heterozygous mouse (right panel). (B) Disruption score of the thyroid gland architecture in the 6 genotypes. (C) Oncocytic score in the 6 genotypes. (D) Serum total T4 levels in the 6 genotypes: lack of LMP2 ameliorates the hypothyroidism typical of *thyr*-IFNγ transgenics. (E) Body weight of male (left panel) and female (right panel) mice in the 6 genotypes: lack of LMP2 ameliorates growth retardation typical of *thyr*-IFNγ transgenic mice.

To assess genetically the effects of immunoproteasome blockade, we crossed *thyr*-IFNγ transgenic mice to mice lacking either LMP2 or LMP7. *Thyr*-IFNγ transgenic - LMP2 knockout mice showed a significantly improved thyroid morphology, with minimal architectural disruption (Figure 4A left panel, and 4B), and a limited number of oncocytes (Figure 4A middle panel, and 4C). Their thyrocytes resembled those of wild type littermates (Figure 4A middle panel). The phenotype was intermediate in *thyr*-IFNγ transgenic mice having one copy of LMP2 (Figure 4A right panel). The scattered mononuclear infiltration typical of *thyr*-IFNγ transgenic mice, instead, was not affected by the absence of LMP2, and lymphocytes could still be seen in the thyroid interstitium of *thyr*-IFNγ transgenic-LMP2 knockout mice (Figure 4A middle panel). Overall, these results suggest that LMP2 is directly involved in the formation of oncocyte initiated by IFNγ. During these studies we also noted that thyroid follicular cells from mice lacking only LMP2 were flatter than those observed in wild type controls (Figure S2A), suggesting an effect of LMP2 on thyroid hormonogenesis. In contrast to LMP2, lack of LMP7 did not improve the disrupted thyroidal morphology or the oncocytic phenotype seen in *thyr*-IFNγ transgenic mice (Figure S2B).

### Genetic Deletion of LMP2 Corrects the Hypothyroidism and Growth Defect Induced by IFNγ

We have previously reported that *thyr*-IFNγ transgenic mice develop primary hypothyroidism (low T4, high TSH, and low iodine uptake) [5], goiter [6], and growth defect [6]. When crossed to LMP2 deficient mice, the *thyr*-IFNγ double mutant mice corrected the hypothyroidism (Figure 4D, sixth group) and restored a normal body weight in both males (Figure 4E left panel) and females (Figure 4E right panel). These ameliorations were intermediate in *thyr*-IFNγ transgenics with a single copy of LMP2 (Figure 4A right panel, and 4B fifth group). The results indicate that LMP2 is involved not only in oncocyte formation but also in the development of hypothyroidism and growth defect initiated by IFNγ. LMP2 deficiency by itself had no influence on circulating T4 levels (Figure 4D, third group) or growth (Figure 4E, third group).

Thyroid accumulation of radioactive iodine in *thyr*-IFNγ transgenics and *thyr*-IFNγ transgenic-LMP2 knockout mice (Figure S3, third and fourth group) was significantly lower than that of wild type controls (Figure S3, first group). Mice lacking only LMP2 (Figure S3, second group) also had a significantly lower radioiodine accumulation than wild type controls, in keeping with the flattened thyrocyte morphology previously described (Figure S2A). LMP2 thus emerges as an important regulator of thyroid structure and function, capable of modulating iodine accumulation independently of IFNγ.

### LMP2 Is Expressed in Human Thyroid Oncocytes (Hürthle Cells)

**Figure 5:**
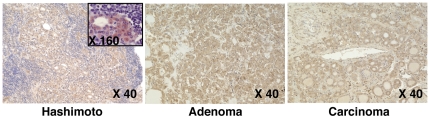
LMP2 expression in human thyroid. LMP2 expression marks Hürthle cells in patients with Hashimoto thyroiditis (left panel) Hürthle cell adenoma (middle panel), or Hürthle cell carcinoma (right panel). The inset on the left panel shows that LMP2 is expressed specifically in Hürthle cells (oncocytes) surrounded by the lymphocytic infiltration.

Given the key role of LMP2 in the induction of mouse oncocytes, we assessed whether LMP2 was also expressed in archival thyroid glands removed from patients with Hashimoto thyroiditis, Graves disease, Hürthle cell adenoma, or Hürthle cell carcinoma. LMP2 was expressed in all 19 Hashimoto thyroiditis specimens (Table 2) with mention of Hürthle cells and in most of those (5 of 6) where Hürthle cells were not mentioned by the pathologist. LMP2 staining in Hashimoto thyroiditis was diffuse throughout the section (Figure 5, left panel) and of strong intensity (Figure 5, left panel inset), often useful to spot oncocytes in the midst of the diffuse mononuclear cell infiltration typical of this disease. LMP2 was also expressed in Hürthle cell adenomas (Figure 5, middle panel) and carcinoma (Figure 5, right panel), although levels varied greatly among patients and different areas of the same specimen (Figure S4A). LMP7 expression followed that of LMP2 in Hashimoto thyroiditis, Hürthle cell adenoma, and carcinoma (Figure S4B).

**Table 2:** Distribution and intensity of LMP2 expression by immunohistochemistry in Hürthle cells present in Hashimoto thyroiditis, Hürthle cell adenoma, Hürthle cell carcinoma, and Graves disease.

	strong LMP2 positive	mild LMP2 positive	no detectable LMP2	total
Hashimoto thyroiditis with mention of Hürthle cells	18 (95%)	1	0	19
Hashimoto thyroiditis without mention of Hürthle cells	3 (50%)	2	1*	6
Hürthle cell adenoma	5 (55%)	4	0	9
Hürthle cell carcinoma	3 (43%)	4	0	7
Graves disease	0	1	1	2

Autoimmune Hürthle cells strongly express LMP2.

* This thyroid pathological specimen contained only infiltrating lymphocytes and no thyroid cells.

Overall the results suggest that oncocytes (Hürthle cells) arise in response to a chronic inflammatory milieu within the thyroid gland, forming a spectrum of morphological variants, with an “autoimmune Hürthle cell” at one end and a “neoplastic Hürthle cell” at the other end of the spectrum.

## Discussion

The study reports that LMP2, an immunoproteasome subunit, is involved in the generation of oncocytes and hypothyroidism in a mouse model of Hashimoto thyroiditis, and that is also expressed in human oncocyte (Hürthle cell) lesions.

The role of LMP2 in the pathogenesis of autoimmune diseases has been explored by a limited number of studies with conflicting results. Some studies have positively linked LMP2 to autoimmunity. For example, polymorphisms in the LMP2 have been associated with autoimmune diseases like ankylosing spondylitis [10], vitiligo [11], and psoriasis [12]. In addition, circulating levels of LMP2 [13] and antibodies to LMP2 [14] are increased in patients with connective tissues disorders like systemic lupus erythematosus and Sjögren syndrome [15], [16]. On the other hand, one group has shown that the lack of splenic LMP2 is linked to development of type 1 diabetes in the NOD mouse [17], [18], a report that has stimulated some controversy [19], [20]. It is likely that the tissue levels of LMP2 change throughout the course of the autoimmune process in a tissue-specific manner. For example, Vives-Pi and colleagues, studying eight patients with Graves disease patients and four with type 1 diabetes, reported that thyroid glands expressed high but variable levels of LMP2 and LMP7, whereas pancreases had levels similar to those found in healthy controls [21]. LMP2 increase did not always correspond to a similar LMP7 increase, in keeping with the notion that these two subunits are not always assembled together [22] in the immunoproteasome, contrary to the pairing of LMP2 and LMP10 [23]. Our finding that only the lack of LMP2, but not LMP7, ameliorated diseases in *thyr-*IFNγ transgenic mice supports these observations.

In addition to demonstrating at the molecular and tissue levels that LMP2 is a marker of oncocytes (Hürthle cells) in Hashimoto thyroiditis, this study also reports that LMP2 is increased in thyroid cancer. In patients with Hürthle cell adenomas or carcinomas the expression of LMP2 was clear, although more variable than that observed in Hashimoto thyroiditis. The variability could be due to fine gradations in the oncocytic phenotype: although oncocytes are defined by the combination of morphological features, these features form a continuous spectrum, leading us to hypothesize that the oncocyte emerging in Hashimoto thyroiditis (“autoimmune Hürthle cell) might be different from those found in cancer (“neoplastic Hürthle cell”). This hypothesis remains to be tested. Further studies are also needed to assess whether LMP2 levels correlate with clinical outcomes in patients with Hürthle cell carcinoma.

LMP2 was used in this study as a therapeutic target to control with MLN-273 the oncocytes present in transgenic mice over-expressing IFNγ in the thyroid gland. MLN-273 (PS-273) is an emerging boronic acid proteasome inhibitor similar to Velcade (bortezomib or PS-341) [24], a drug used in the treatment of several cancers like multiple myeloma [25], and more recently inflammatory [26] and autoimmune [27] diseases. MLN-273, like Velcade, blocks the constitutive proteasome (β5 subunit) and the immunoproteasome [28]. In vitro, MLN-273 effectively blocks the development of Plasmodium parasites [29] and Mycobacterium tuberculosis [30]. In vivo, MLN-273 halts the degradation of dystrophin and dystrophin-associated proteins in a mouse model of Duchenne muscular dystrophy [31]. It also improves the glomerular filtration rate in a pig model of hypercholesterolemia [32], although there is disagreement on whether targeting the proteasome actually worsens atherosclerosis [33]–[35]. It remains to be tested whether proteasome inhibition is effective in the treatment of Hürthle cell carcinomas. As new inhibitors (like IPSI-001) are being developed that preferentially target the immunoproteasome rather than constitutive proteasome [28], it is possible that this treatment modality will extend to a larger number of autoimmune diseases. The treatment might be also applicable to oncocytic lesions found in other organs, like kidneys, salivary or adrenal glands.

Interestingly, the lack of LMP2 ameliorated the hypothyroidism seen in *thyr-*IFNγ transgenic mice, providing a novel explanation for the pathogenesis of hypothyroidism typically found in patients with autoimmune thyroiditis. Architectural disruption of the thyroid gland by infiltrating lymphocytes and apoptosis of thyrocytes have traditionally been considered the cause of hypothyroidism in autoimmune thyroiditis, but they are not the target of therapeutic interventions, which instead rely on the administration of synthetic thyroxine. Although effective, this symptomatic treatment is unsatisfactory in a discrete percentage of hypothyroid patients, highlighting the need for new treatments based on disease pathogenesis. *Thyr-*IFNγ transgenic mice can, therefore, provide a model to study chronic hypothyroidism, a feature not found in most animal models of autoimmune thyroiditis.

In summary, we report that thyroid oncocytes (Hürthle cells) arise in the thyroid gland in response to a chronic inflammatory milieu concomitant with LMP2 over expression, and suggest they form a spectrum of morphological variants with an “autoimmune Hürthle cell” at one end and a “neoplastic Hürthle cell” at the other end of the spectrum. Considering increasing reports of the association between Hashimoto thyroiditis and thyroid cancer [36], we strongly propose that LMP2 might be a potential therapeutic target for the protective treatment of oncocytic lesions and hypothyroidism as well.

## Materials and Methods

### Mice

The study analyzed a total of 366 mice: 216 genetically modified, and 150 C57BL/6 wild type littermate controls. The genetically modified mice included the following genotypes, all on the BL/6 background: *thyr*-IFNγ transgenics (N = 95) (a model previously generated in our laboratory to express murine IFNγ specifically in thyroid follicular cells [5]), LMP2 knockout (N = 29); LMP2 heterozygous (N = 19); LMP2 knockout/IFNγ transgenic (N = 46); LMP2 heterozygous/IFNγ transgenic (N = 19); LMP7 knockout/IFNγ transgenic (N = 5); and STAT1 knockout/IFNγ transgenic (N = 3). The *thyr*-IFNγ transgenic mice were generated as previously described. They express the murine IFNγ gene under control of the rat thyroglobulin promoter, to support transcription specifically in thyroid follicular cells. LMP2 knockout mice and LMP7 knockout mice (C57BL/6 background) were kindly gifted from Dr. Whitton (The Scripps Research Institute, La Jolla, CA) [37]. The following primers were used for genotyping for LMP2 or LMP7 knockout mice, LMP2-2 primer: CCCGTGTCCCTCCGAGATAC and LMP2-4 primer: GGGATCCAGGACCAGGAAAG to detect endogenous *Psmb9* (LMP2) gene, LMP7A primer: TCTATGGTGGCATCACTATGTT and LMP7B primer: TGGTACACTGTGGGCGATTGAG to detect endogenous *Psmb8* (LMP7) gene, NEO A primer: CTTGGGTGGAGAGGCTATTC and NEO B primer: AGGTGAGATGACAGGAGATC to detect mutant gene containing *neo* gene. STAT1 knockout mice, also on the C57BL/6 background, were kindly donated by Dr. David Levy (New York University Medical Center, New York, NY) [38]. The following primers were used for genotyping for LMP2 or LMP7 knockout mice, Stat P1 primer: GAGATAATTCACAAAATCAGAGAG and Stat P2 primer: CTGATCCAGGCAGGCGTTG to detect endogenous *Stat1* gene, Stat P1 primer and Stat P3 primer: TAATGTTTCATAGTTGGATATCAT to detect mutant gene. All mice were bred in specific pathogen-free facilities at Johns Hopkins University following protocols conformed to the JHU Animal Care and Use Committee guidelines. For some experiments, mice were weighed at five-week old.

### Human Thyroid Glands

Thyroid samples (N = 43) for morphological and immunohistochemical studies were obtained from the archival surgical pathology specimens maintained in The Johns Hopkins Pathology Department, using a protocol approved by the Institutional Review Board. Samples included patients with Hashimoto thyroiditis (N = 25), Graves disease (N = 2), Hürthle cell adenoma (N = 9), and Hürthle cell carcinoma (N = 7).

### Histopathology of Thyroid

After euthanasia, mouse tracheas with attached thyroids were removed and fixed for 48 hours in the zinc-based Beckstead's solution or buffer-saturated formalin. The specimen was then processed and embedded in paraffin. Six to 8 non sequential sections (5 µm thick) were cut from the tissue block and stained with hematoxylin and eosin (H&E). The same tissue block used for H&E histopathology was used to cut sections for immunohistochemistry.

### TUNEL Staining to Detect Apoptosis in Mouse Thyroid Glands

Formalin-fixed thyroid sections were deparaffinized in xylene, rehydrated in decreasing concentrations of ethanol, and washed in phosphate buffer saline (PBS). Sections were then incubated at room temperature in proteinase K (20 µg/ml for 15 min), washed in distilled water, and then incubated in hydrogen peroxide (0.3% for 10 min). TUNEL staining was performed using the ApopTag kit (Chemicon, Temecula, CA). Sections were first equilibrated in the kit buffer and then incubated for 1 hour at 37°C in a humidified chamber with terminal deoxynucleotidyl transferase conjugated to digoxigenin. Sections were then placed in a Coplin jar containing working strength stop/wash buffer, agitated for 15 seconds, and incubated for 10 min at RT. After washing in PBS, an anti-digoxigenin antibody conjugated to peroxidase was applied for 30 min at RT. After washing, apoptosis-specific color was obtained using diaminobenzidine, and overall morphology using Eosin Y Alcoholic (Richard-Allan Scientific, Kalamazoo, MI).

### Electron Microscopy of Mouse Thyroid Glands

Thyroid glands were fixed for 1 hour at room temperature in 0.1 M cacodylate buffer pH 7.2, supplemented with 3 mM CaCl_2_, 2% glutaraldehyde, and 4% paraformaldehyde. Glands were then post-fixed for 1 hour at 4°C in cacodylate buffer supplemented with 3 mM CaCl_2_ and 2% osmium tetroxide. Glands were then washed for 30 min in 2% uranyl acetate, and dehydrated first with increasing concentrations of ethanol (50%, 70%, 90% and 100%) and then with propylene oxide. Glands were then infiltrated with a propylene oxide-Epon mixture and embedded flat in Epon. Ultra-thin sections were cut and analyzed for electron microscopy using a Phillips CM12 (S)TEM microscope.

### Long SAGE (Serial Analysis of Gene Expression) of CD45 Negative Mouse Thyrocytes

Thyroid lobes were collected from 3 *thyr*-IFNγ transgenic female mice, and 16 wild type female littermates. Lobes were incubated immediately after dissection for 26 hours at 4°C in Eagle's minimal essential medium, supplemented with 1.2 U/mL dispase II (Roche Applied Science, Indianapolis, IN) and 0.25 U/mL collagenase II (Sigma, St. Louis, MO), to ensure a thorough diffusion of the enzymes into the tissue. Lobes were then digested for 20 min at 37°C in a shaking water bath, and then briefly in cold PBS containing 2 mM EDTA and 1% bovine serum albumin to stop the digestion. After one wash, thyroid cells were first passed through 70 µM mesh filter, to remove large tissue debris, and then incubated for 15 minutes at 4°C with anti-CD45 paramagnetic microbeads (Miltenyi Biotech, Auburn, CA). After one wash, the mixture was applied to a MS column (Miltenyi Biotech) in a magnetic separator: the CD45 negative fraction (composed mainly of thyrocytes, parathyroid cells, and endothelial cells) was eluted by the addition of phosphate-buffered saline, supplemented with 2 mM EDTA and 0.5% bovine serum albumin. The magnetic separation was repeated once. CD45 negative thyroid cells were counted with trypan blue and used to extract mRNA and perform Long SAGE as described by Saha S *et al*. [39]. The SAGE sequencing data are available from the Gene Expression Omnibus repository of the National Center for Biotechnology Information (http://www.ncbi.nlm.nih.gov/geo), using series record number GSE15114.

### Expression of Immunoproteasome Subunit mRNA in Mouse Thyroids by Semi-Quantitative Reverse Transcriptase PCR

Thyroid lobes from 1-month-old *thyr*-IFNγ transgenics (N = 3) and wild type littermates (N = 3) were mechanically homogenized to extract mRNA using oligo (dT)25 magnetic beads (Invitrogen, Carlsbad, CA). Following DNase I (Invitrogen) treatment, mRNA was reverse transcribed into cDNA using Superscript II RNase H^-^ reverse transcriptase (Invitrogen). The five mouse immunoproteasome subunits using the following specific primers: PA28α forward primer 5′-CAAGCCAAGGTGGATGTGTTCC-3′ and reverse primer 5′-GATCATTCCCTTGGTTTCTCCACG-3′, PA28β forward primer 5′-GCAGGAGAAGGAAGTCCCTA-3′ and reverse primer 5′-GATGGCTTTTCTTCACCCTTCGG-3′, LMP2 forward primer 5′-ATGCTGCGGGCAGGAGCACCTACCG-3′ and reverse primer 5′-TCACTCATCGTAGAATTTTGGCAGCT-3′, LMP7 forward primer 5′-ATGGCGTTACTGGATCTGTGCGGTGC-3′ and reverse primer 5′-TCACAGAGCGGCCTCTCCGTACTTGT-3′, LMP10 forward primer 5′-AGGAATGCGTCCTTGGAACACG-3′ and reverse primer 5′-TCAATGCTCTCTGCAGCTTGGC-3′. Mouse G3PDH was amplified as quantitation control using forward primer 5′-GCATCTTGGGCTACACTGAG-3′ and the reverse primer 5′-TCTCTTGCTCAGTGTCCTTG-3′. The cDNA from each proteasome sample was sequentially diluted and then amplified for GAPDH. The dilutions that gave similar GAPDH intensities were used for amplification of the proteasome subunits.

### Expression of Immunoproteasome Subunit Proteins in Mouse and Human Thyroids by Immunohistochemistry

Sections were deparaffinized with xylene, rehydrated with decreasing concentrations of ethyl alcohol and finally PBS. Formalin-fixed sections (human section) were, then, treated with target retrieval solution (DAKO, Carpinteria, CA) for 10 min in steamer; no target retrieval treatment for zinc-based Beckstead's solution-fixed mouse tissues. After cool down and washing with PBS, endogenous peroxidase was blocked by treating the sections with 3% H_2_O_2_ in PBS for 30 min at room temperature. After blocking aspecific binding with 5% normal goat serum, sections were incubated with the primary antibody; rabbit anti proteasome subunit (LMP2, LMP7, LMP10, PA28α, and PA28β; Biomol International, L.P., Plymouth Meeting, PA), in PBS supplemented with 1% bovine serum albumin, and incubated overnight at 4°C in a humid chamber. Primary antibody was diluted for human thyroid sections as following; LMP2 (1∶200), LMP7 (1∶2,000), LMP10 (1∶1,000), PA28α (1∶4,000), PA28β (1∶20,000). Primary antibody was diluted for mouse sections as following; LMP2 (1∶400), LMP7 (1∶10,000), LMP10 (1∶4,000), PA28α (1∶12,000), PA28β (1∶40,000).

After washing, the biotin-conjugated secondary antibody (affinity purified goat anti-rabbit IgG, from DAKO, diluted 1∶1000 in PBS-1%BSA) was incubated for 1 hr at room temperature. After rinsing, peroxidase-conjugated streptavidin (DAKO) was diluted 1∶500 in PBS and incubated for 30 min at RT. Finally, sections were incubated for 5 min with NovaRED substrate or DAB substrate (both from Vector Laboratories, Burlingame, CA) for color development and then rinsed in distilled water. Sections were counter-stained with hematoxylin (Vector Laboratories).

### Proteasome Inhibitor Administration

To block pharmacologically the function of the proteasome, *thyr*-IFNγ transgenic mice and wild type littermates were injected i.p with MLN-273. This compound, a gift from Millennium Pharmaceutical Inc. (Cambridge, MA), blocks like Velcade (Bortezomib) the constitutive proteasome (β5 subunit) and the immunoproteasome [28]. MLN-273 was formulated as 1 part (8.3%) active compound and 11 parts (91.7%) mannitol vehicle. Mice received MLN-273 or mannitol control twice a week for 2 weeks, then rested for a week, and then were injected again until reaching cumulative formulated doses of 1.2, 2.0, 2.5, 3.6, 4.8, and 6.0 mg/Kg (Figure 3A).

### Assessment of Thyroid Function by Serum Total T4 Levels

Serum total thyroxine (T4) was determined by a competitive RIA (Diasorin, Stillwater, MN), according to the manufacturer's instructions. Briefly, ten µl of serum were added to plastic tubes coated with a monoclonal antibody directed against T4. Then, 1 ml of tracer buffer containing ^125^I-T4 was added to the tubes and the solution incubated at room temperature for 45 min. Tubes were then decanted and counted for 1 min in a gamma counter (Isodata-520, from Titertek, Huntsville, AL). Total T_4_ concentrations were extrapolated from a standard curve generated using six T_4_ standards ranging from 0 to 20 µg/dL.

### Radioactive Iodine Uptake (RAIU)

Iodine uptake was determined using radioactive iodine. Mice were fed with iodine-deficient diet (TD95007 from HARLAN TEKLAD, Indianapolis, IN) for 3 weeks. Mice were injected with 10 µCi of ^125^I (carrier-free, Amersham) intraperitoneally. Mice were sacrificed 4 hours after injection of radioactive iodine, and thyroid lobes and muscle (negative control) were excised, weighed and counted using a gamma counter. Iodine uptake was normalized dividing the count by the weight of each tissue.

### Detection of Thyroidal Gene Expression by Northern Hybridization

After thyroid lobes were excised and homogenated in TRIZOL (Invitrogen, Carlsbad, CA), total RNA was extracted following manufacture protocol. 10 µg of total RNA was separated by 1% agarose gel electrophoresis and analyzed by Northern blot for the expression of LMP2. The signal from G3PDH was also obtained to adjust for the amount of RNA loaded in each lane; G3PDH probe was synthesized using pTRI-GAPDH-Mouse (Ambion, Austin, TX). Probe signals were quantified using the BAS-1500 Bioimaging Analyzer (Fuji Photo Film Co., Japan).

### Statistical Analysis

Differences among the six genotypes were assessed using the non-parametric Kruskal-Wallis test, followed by Wilcoxon sing-rank test for the pairwise comparisons. All analyses were performed using Stata statistical software, release 10 (from Stata Corp., College Station, TX).

## Supporting Information

Figure S1 (A) LMP2 and LMP10 protein expression by immunohistochemistry in *thyr*-IFNγ transgenic-STAT1 knockout mice: lack of STAT1 abolishes the expression of both immunoproteasome subunits, as compared to that found in *thyr*-IFNγ transgenic-STAT1 wild type controls (insets). (B) LMP2 RNA expression by Northern blotting in Fisher rat thyroid follicular cells (FRTL-5 line) stimulated with IFNγ. Total RNA was extracted from FRTL-5 cells after 0, 3, 12, 24, 48 or 72 hours of IFNγ stimulation, and hybridized with a rat LMP2 cDNA probe by mRNA expression was assessed by Northern hybridization. IFNγ strongly induced LMP2 expression, beginning at 12 hours post-stimulation and plateauing at 48 hours. No oncocytic changes were seen morphologically in the thyroid cells during these culture time points.
(1.75 MB TIF)

Figure S2 (A) Thyroidal morphology of mice lacking just LMP2: the thyroid architecture is preserved (left panel), but thyrocytes are flatter (right panel) than wild type thyrocytes (right panel, inset). (B) Thyroid morphology of *thyr*-IFNγ transgenic mice lacking LMP7: the absence of LMP7 has no effect on the oncocytic phenotype induced by IFNγ.
(9.59 MB TIF)

Figure S3 Radioactive iodine accumulation in thyroid. The radioactive iodine accumulation is decreased in mice lacking LMP2, in *thyr*-IFNγ transgenic mice, and in *thyr*-IFNγ transgenic mice lacking LMP2. No significant difference was observed among LMP2 knockout, *thyr*-IFNγ transgenics, and *thyr*-IFNγ transgenic/LMP2 knockout mice.
(0.27 MB TIF)

Figure S4 Histological analysis of human Hürthle cells. (A) LMP2 expression varies in different areas of Hürthle cell adenomas: expression is lower (left panel, inset) in areas with more complex histopathology (left panel) than in areas with a more uniform appearance (right panel). (B) LMP7 expression in Hashimoto thyroiditis (left panel), Hürthle cell adenoma (middle panel), and Hürthle cell carcinoma (right panel).
(3.13 MB TIF)

Table S1 Top 10 genes expressed in *thyr*-IFNγ transgenic mouse thyrocytes (total tag # is 43,718).
(0.06 MB DOC)

Table S2 Top 10 genes expressed only in *thyr*-IFNγ transgenic mouse thyrocytes.
(0.04 MB DOC)

Table S3 Top 10 genes expressed in wild-type mouse thyrocytes (total tag # is 43,908).
(0.05 MB DOC)

Table S4 Top 10 genes expressed only in wild-type mouse thyrocytes.
(0.03 MB DOC)
