# Supplementary figures and images for: Immunoproteasome Overexpression Underlies the Pathogenesis of Thyroid Oncocytes and Primary Hypothyroidism: Studies in Humans and Mice
Source: PLoS One. 2009 Nov 17;4(11):e7857. doi: 10.1371/journal.pone.0007857 (PMC2773418; doi:10.1371/journal.pone.0007857)

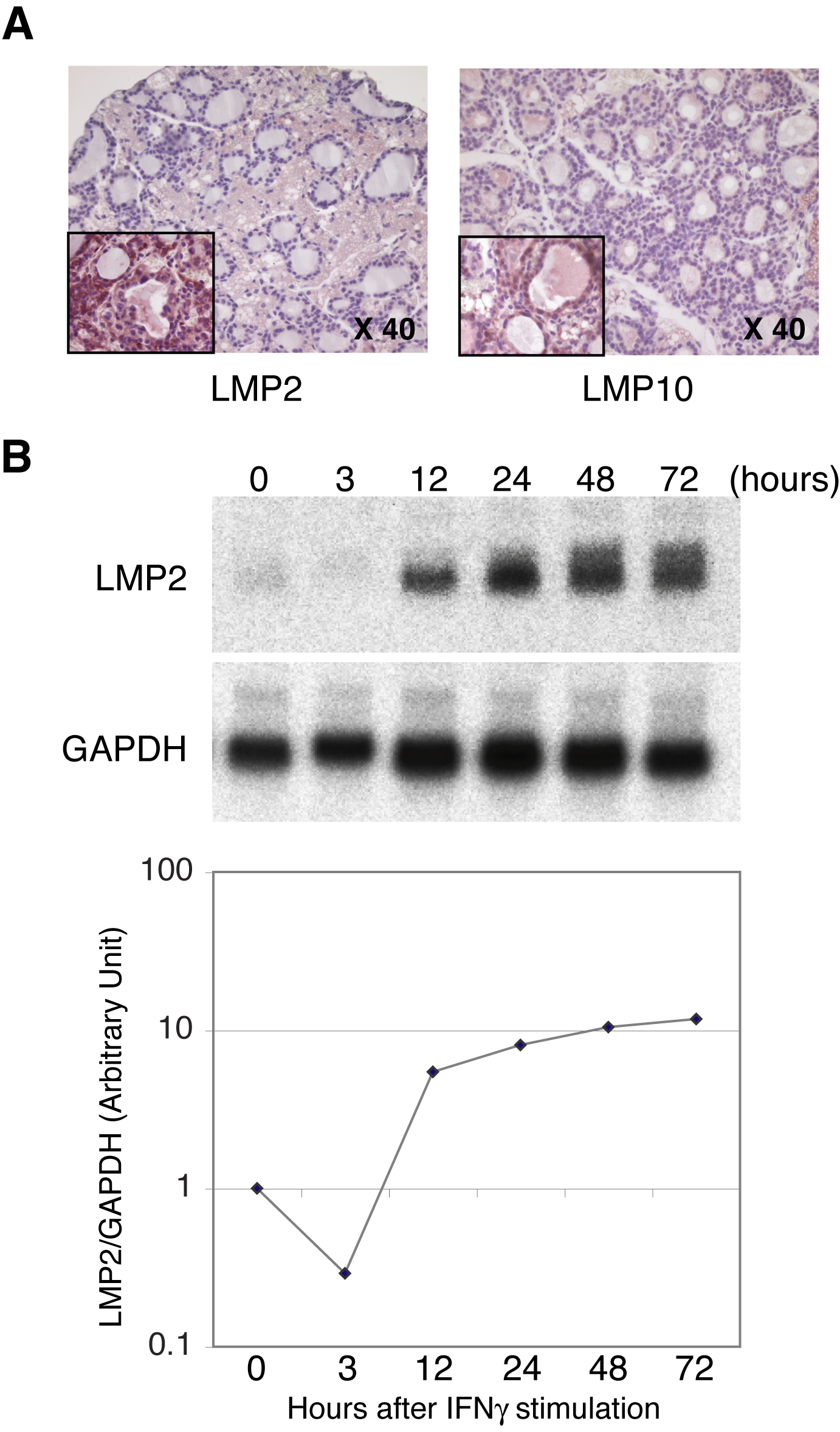

Supplement: Figure S1 — (A) LMP2 and LMP10 protein expression by immunohistochemistry in thyr-IFNγ transgenic-STAT1 knockout mice: lack of STAT1 abolishes the expression of both immunoproteasome subunits, as compared to that found in thyr-IFNγ transgenic-STAT1 wild type controls (insets). (B) LMP2 RNA expression by Northern blotting in Fisher rat thyroid follicular cells (FRTL-5 line) stimulated with IFNγ. Total RNA was extracted from FRTL-5 cells after 0, 3, 12, 24, 48 or 72 hours of IFNγ stimulation, and hybridized with a rat LMP2 cDNA probe by mRNA expression was assessed by Northern hybridization. IFNγ strongly induced LMP2 expression, beginning at 12 hours post-stimulation and plateauing at 48 hours. No oncocytic changes were seen morphologically in the thyroid cells during these culture time points. (1.75 MB TIF) [file pone.0007857.s001.tif]

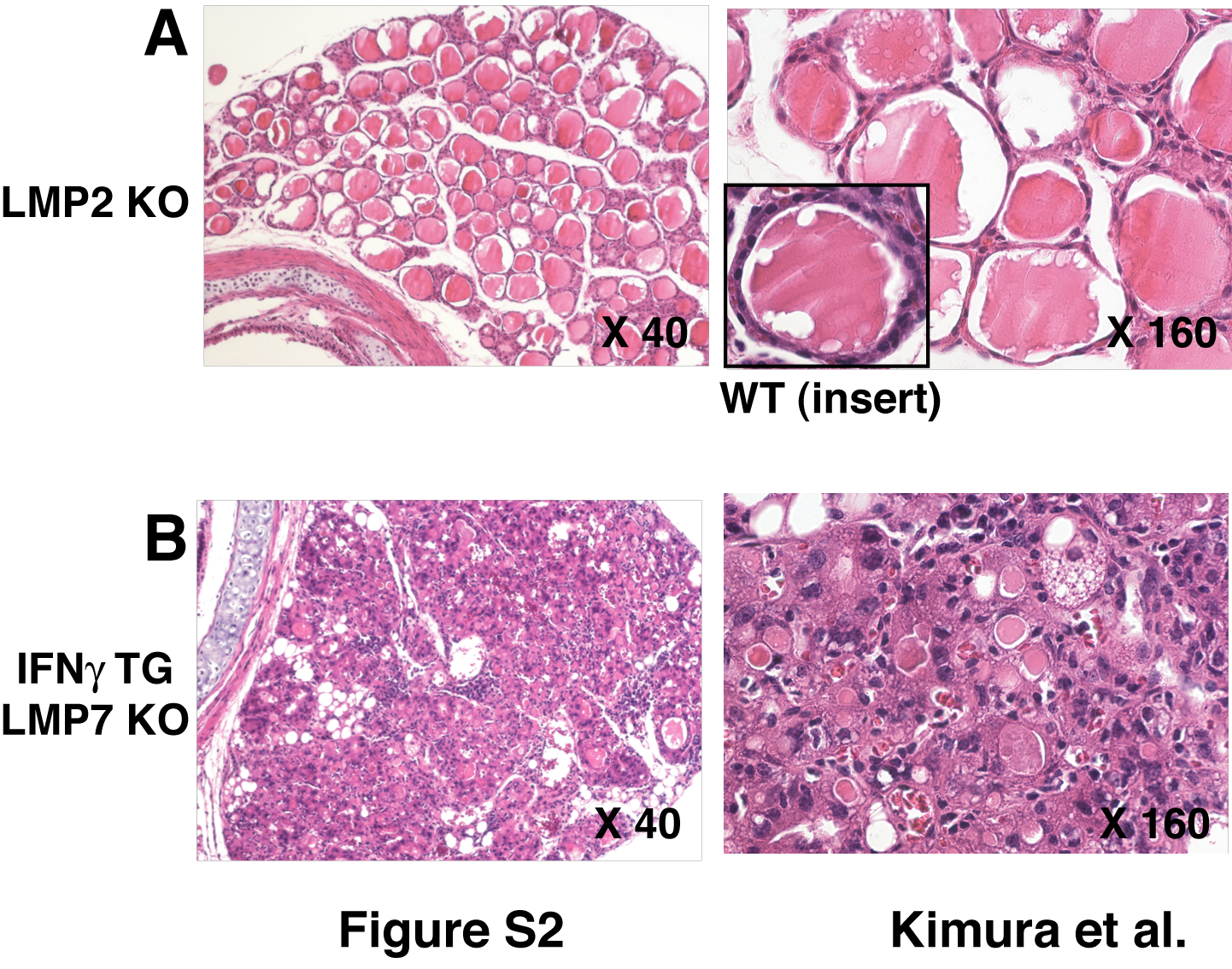

Supplement: Figure S2 — (A) Thyroidal morphology of mice lacking just LMP2: the thyroid architecture is preserved (left panel), but thyrocytes are flatter (right panel) than wild type thyrocytes (right panel, inset). (B) Thyroid morphology of thyr-IFNγ transgenic mice lacking LMP7: the absence of LMP7 has no effect on the oncocytic phenotype induced by IFNγ. (9.59 MB TIF) [file pone.0007857.s002.tif]

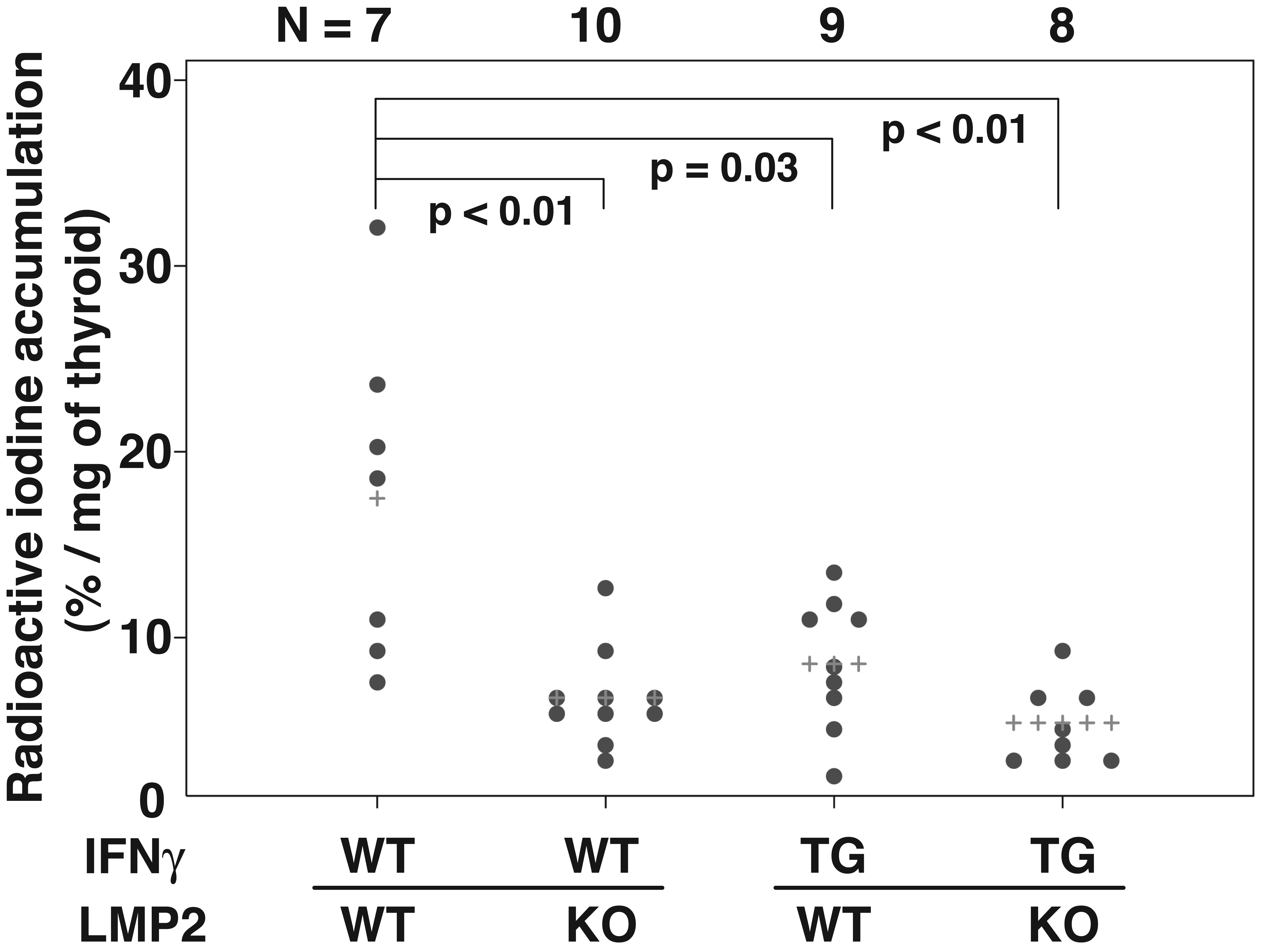

Supplement: Figure S3 — Radioactive iodine accumulation in thyroid. The radioactive iodine accumulation is decreased in mice lacking LMP2, in thyr-IFNγ transgenic mice, and in thyr-IFNγ transgenic mice lacking LMP2. No significant difference was observed among LMP2 knockout, thyr-IFNγ transgenics, and thyr-IFNγ transgenic/LMP2 knockout mice. (0.27 MB TIF) [file pone.0007857.s003.tif]

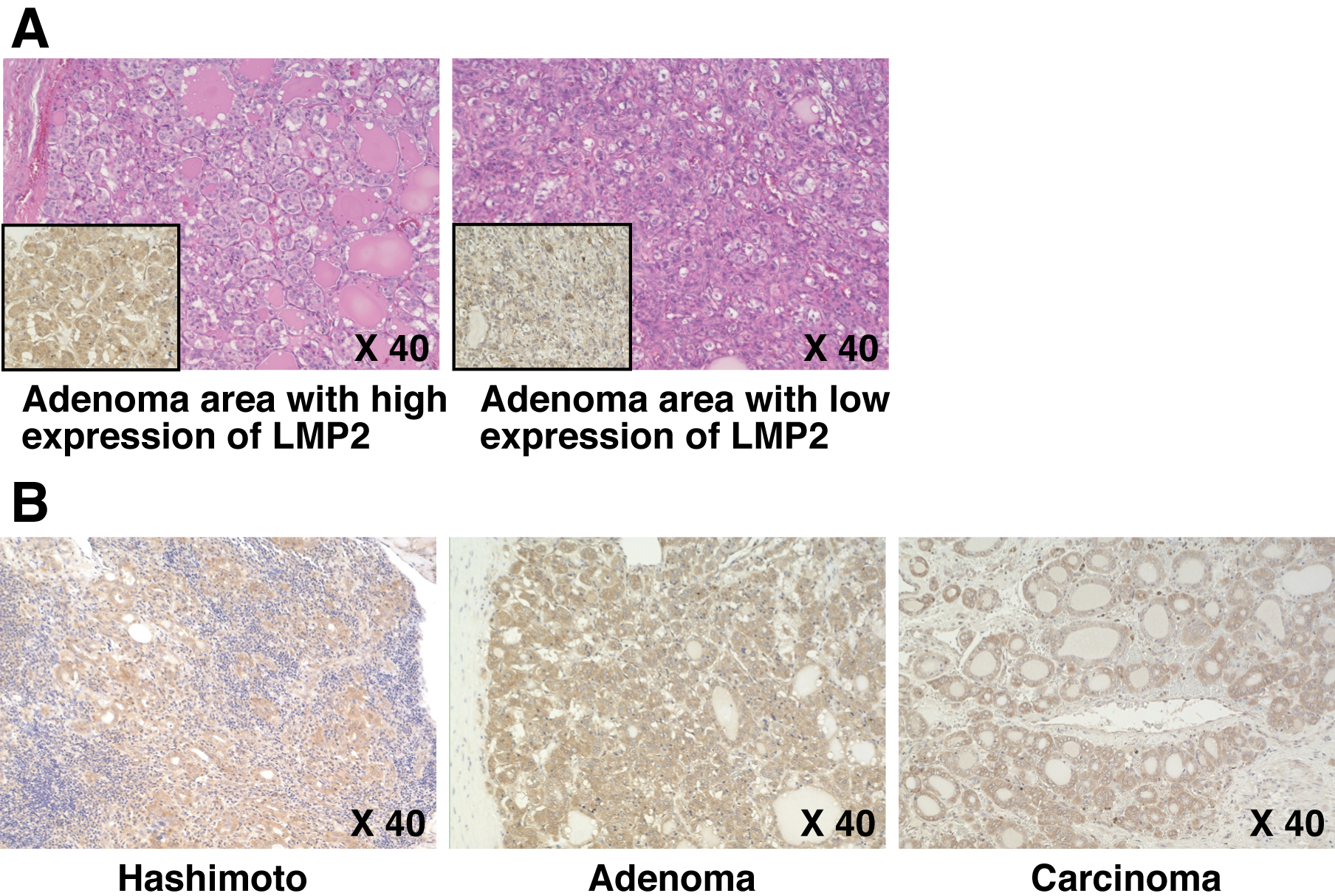

Supplement: Figure S4 — Histological analysis of human Hürthle cells. (A) LMP2 expression varies in different areas of Hürthle cell adenomas: expression is lower (left panel, inset) in areas with more complex histopathology (left panel) than in areas with a more uniform appearance (right panel). (B) LMP7 expression in Hashimoto thyroiditis (left panel), Hürthle cell adenoma (middle panel), and Hürthle cell carcinoma (right panel). (3.13 MB TIF) [file pone.0007857.s004.tif]
