# Supplementary material for: Immunoproteasome Overexpression Underlies the Pathogenesis of Thyroid Oncocytes and Primary Hypothyroidism: Studies in Humans and Mice
Source: PLoS One. 2009 Nov 17;4(11):e7857. doi: 10.1371/journal.pone.0007857 (PMC2773418; doi:10.1371/journal.pone.0007857)
Supplement: Table S2 — Top 10 genes expressed only in thyr-IFNγ transgenic mouse thyrocytes. (0.04 MB DOC) [file pone.0007857.s006.doc]

Table S2: Top 10 genes expressed only in *thyr*-IFNγ transgenic mouse thyrocytes.

| Rank | Gene Description | Uni Gene # | # of tags /10,000  (rounded) |
| --- | --- | --- | --- |
| 1 | H2-Aa Histocompatibility 2, class II antigen A, alpha, mRNA | Mm.235338 | 35 |
| 2 | H2-Ab1 Histocompatibility 2, class II antigen A, beta 1, mRNA | Mm.254067 | 30 |
| 3 | H2-D1 Histocompatibility 2, D region, mRNA | Mm.33263 | 8 |
| 4 | Indo Indoleamine-pyrrole 2,3 dioxygenase (Indo), mRNA | Mm.392 | 7 |
| 5 | Iigp1 Interferon inducible GTPase 1, mRNA | Mm.261140 | 6 |
| 6 | Ribosomal protein L37a, mRNA | Mm.21529 | 6 |
|  | Rpl37a Ribosomal protein L37a (Rpl37a), mRNA | Mm.379003 |  |
| 6 | Ribosomal protein S28, mRNA | Mm.200920 | 6 |
|  | Rps28 Ribosomal protein S28 (Rps28), mRNA | Mm.371603 |  |
| 8 | Aplp2 Amyloid beta (A4) precursor-like protein 2, mRNA | Mm.19133 | 4 |
| 8 | Macrophage activation 2 like, mRNA | Mm.275893 | 4 |
|  | Mpa2l Macrophage activation 2 like (Mpa2l), mRNA | Mm.379280 |  |
| 8 | Blastocyst cDNA, RIKEN full-length enriched library, clone:I1C0003E23 product:Ribosomal protein S13, full insert sequence | Mm.14798 | 4 |
|  | Rps13 Ribosomal protein S13, mRNA | Mm.345443 |  |
