# Supplementary material for: Immunoproteasome Overexpression Underlies the Pathogenesis of Thyroid Oncocytes and Primary Hypothyroidism: Studies in Humans and Mice
Source: PLoS One. 2009 Nov 17;4(11):e7857. doi: 10.1371/journal.pone.0007857 (PMC2773418; doi:10.1371/journal.pone.0007857)
Supplement: Table S3 — Top 10 genes expressed in wild-type mouse thyrocytes (total tag # is 43,908). (0.05 MB DOC) [file pone.0007857.s007.doc]

Table S3: Top 10 genes expressed in wild-type mouse thyrocytes (total tag # is 43,908).

| Rank | Gene Description | Uni Gene # | # of tags /10,000  (rounded) |
| --- | --- | --- | --- |
| 1 | Tgn Thyroglobulin (Tgn), mRNA | Mm.12800 | 283 |
| 2 | Tgn Thyroglobulin (Tgn), mRNA | Mm.12800 | 102 |
| 3 | Hbb-b1 Hemoglobin, beta adult major chain, mRNA | Mm.288567 | 62 |
|  | Hbb PREDICTED: similar to Hemoglobin beta-1 chain (B1) (Hemoglobin beta-major chain), mRNA sequence | Mm.387214 |  |
| 4 | Transcribed locus, weakly similar to NP_904337.1 NADH dehydrogenase subunit 4 | Mm.261851 | 57 |
|  | Suclg1 Succinate-CoA ligase, GDP-forming, alpha subunit, mRNA | Mm.29845 |  |
| 5 | Hba-a1 Hemoglobin alpha, adult chain 1 (Hba-a1), mRNA | Mm.196110 | 45 |
|  | H3f3a H3 histone, family 3A, mRNA | Mm.315189 |  |
| 6 | Tgn Thyroglobulin (Tgn), mRNA | Mm.12800 | 43 |
| 7 | Tgn Thyroglobulin (Tgn), mRNA | Mm.12800 | 41 |
| 8 | Elavl1 ELAV (embryonic lethal, abnormal vision, Drosophila)-like 1 (Hu antigen R), mRNA | Mm.119162 | 31 |
|  | Exosc2 Exosome component 2 (Exosc2), mRNA | Mm.150972 |  |
|  | 1300015B04Rik RIKEN cDNA 1300015B04 gene, mRNA | Mm.169261 |  |
|  | Itch Ubiquitin protein ligase (Itch) | Mm.208286 |  |
|  | Cyp2e1 Cytochrome P450, family 2, subfamily e, polypeptide 1, mRNA | Mm.21758 |  |
|  | 5530601H04Rik PREDICTED: hypothetical protein LOC71445, mRNA sequence | Mm.242968 |  |
|  | Acmsd Amino carboxymuconate semialdehyde decarboxylase (Acmsd), mRNA | Mm.25735 |  |
|  | PREDICTED: similar to ZNF43 protein, mRNA sequence | Mm.260056 |  |
|  | Wap Whey acidic protein, mRNA | Mm.268094 |  |
|  | Stat5a Signal transducer and activator of transcription 5B (Stat5b) | Mm.277403 |  |
|  | D19Ertd737e DNA segment, Chr 19, ERATO Doi 737, expressed, mRNA | Mm.290183 |  |
|  | Cklfsf7 Chemokine-like factor super family 7, mRNA | Mm.35600 |  |
|  | Leprel2 Leprecan-like 2, mRNA | Mm.35708 |  |
|  | Fbxo24 PREDICTED: F-box only protein 24, mRNA sequence | Mm.379192 |  |
|  | Irak1 IRAK1-S mRNA for interleukin-1 receptor-associated kinase-1-S | Mm.38241 |  |
|  | Thap1 THAP domain containing, apoptosis associated protein 1, mRNA | Mm.383241 |  |
|  | Gpr21 G protein-coupled receptor 21 (Gpr21), mRNA | Mm.386910 |  |
|  | Ifnar1 Interferon (alpha and beta) receptor 1 (Ifnar1), mRNA | Mm.502 |  |
| 9 | Tgn Thyroglobulin (Tgn), mRNA | Mm.12800 | 29 |
| 10 | 0910001K20Rik LMBR1 domain containing 1, mRNA | Mm.336563 | 26 |
