# Supplementary material for: Immunoproteasome Overexpression Underlies the Pathogenesis of Thyroid Oncocytes and Primary Hypothyroidism: Studies in Humans and Mice
Source: PLoS One. 2009 Nov 17;4(11):e7857. doi: 10.1371/journal.pone.0007857 (PMC2773418; doi:10.1371/journal.pone.0007857)
Supplement: Table S4 — Top 10 genes expressed only in wild-type mouse thyrocytes. (0.03 MB DOC) [file pone.0007857.s008.doc]

Table S4: Top 10 genes expressed only in wild-type mouse thyrocytes.

| Rank | Gene Description | Uni Gene # | # of tags /10,000  (rounded) |
| --- | --- | --- | --- |
| 1 | Tgn Thyroglobulin (Tgn), mRNA | Mm.12800 | 26 |
| 2 | Tgn Thyroglobulin (Tgn), mRNA | Mm.12800 | 21 |
| 3 | Hba-a1 Hemoglobin alpha, adult chain 1 (Hba-a1), mRNA | Mm.196110 | 17 |
| 4 | Hbb-b1 Hemoglobin, beta adult major chain, mRNA | Mm.288567 | 14 |
|  | Hbb PREDICTED: similar to Hemoglobin beta-1 chain (B1) (Hemoglobin beta-major chain), mRNA sequence | Mm.387214 |  |
| 5 | Tgn Thyroglobulin (Tgn), mRNA | Mm.12800 | 8 |
| 6 | Hba-a1 Hemoglobin alpha, adult chain 1 (Hba-a1), mRNA | Mm.196110 | 7 |
|  | cDNA clone IMAGE:6814413 | Mm.21450 |  |
| 7 | Tgn Thyroglobulin (Tgn), mRNA | Mm.12800 | 7 |
| 7 | Fth1 Ferritin heavy chain 1, mRNA | Mm.1776 | 7 |
| 7 | Hba-a1 Hemoglobin alpha, adult chain 1 (Hba-a1), mRNA | Mm.196110 | 7 |
| 7 | Tgn Thyroglobulin (Tgn), mRNA | Mm.12800 | 7 |
